# Effect of Amnioreduction Prior to Emergency Cervical Cerclage on Pregnancy Outcomes in Singleton Pregnancies with Painless Cervical Dilation: A Multicenter Retrospective Study

**DOI:** 10.3390/jcm15145431

**Published:** 2026-07-10

**Authors:** Xiaotian Ni, Yinglin Liu, Xiaolu Nie, Yun Liu, Honglei Ji, Shengyao Lei, Guojun Ma, Chunyan Shi, Tao Duan, Qiong Luo, Ming Liu

**Affiliations:** 1 Department of Obstetrics, Shanghai East Hospital, Tongji University School of Medicine, Shanghai 200123, China; ni_xiaotian@126.com (X.N.); lyun812@126.com (Y.L.); hongleijish@163.com (H.J.); leishengyao1234@163.com (S.L.); maguojun1009@163.com (G.M.); drduantao@126.com (T.D.); 2 Department of Obstetrics & Gynecology, Sun Yat-sen Memorial Hospital, Sun Yat-sen University, Guangzhou 510120, China; lyl-sharry@163.com (Y.L.);; 3 Department of Obstetrics & Gynecology and Reproductive Medicine, Peking University First Hospital, Beijing 100034, China; shichunyan1996@163.com; 4 Department of Obstetrics, Women’s Hospital, School of Medicine, Zhejiang University, Hangzhou 310027, China

**Keywords:** emergency cervical cerclage, amnioreduction, cervical insufficiency, preterm birth, pregnancy outcome, propensity score matching

## Abstract

**Background/Objectives**: Amnioreduction prior to emergency cervical cerclage (ECC) has been proposed as a method to relieve the tension of the protruding amniotic membrane, thereby facilitating its intact retraction back into the uterine cavity. However, the effect of this procedure on pregnancy outcomes remains unclear. This study aims to evaluate the effect of amnioreduction on pregnancy outcomes in patients who received ECC during the second trimester. **Methods**: We conducted a retrospective analysis of the characteristics and pregnancy outcomes of patients who underwent ECC across four institutions between 1 July 2021 and 31 December 2024. Pregnancies were classified into the amnioreduction group and the non-amnioreduction group based on whether amnioreduction was performed prior to the ECC. The gestational age (GA) at delivery was the primary outcome measure. A multivariate linear regression model was employed to identify factors associated with GA at delivery. Additionally, a propensity score matching model was constructed for sensitivity analysis. A subgroup analysis was also conducted for pregnancies with cervical dilation ≥ 4 cm to evaluate the effect of amnioreduction in this specific subpopulation. **Results**: A total of 406 pregnancies were analyzed, and the results indicated no significant differences between the amnioreduction group and the non-amnioreduction group regarding GA at delivery (30.4 (25.7, 35.9) vs. 31.2 (26.0, 37.0) weeks, *p* = 0.655) or pregnancy latency (days from cerclage to delivery: 49.0 (15.0, 87.5) vs. 57.0 (18.0, 92.3) days, *p* = 0.397). The rates of preterm birth at <28 weeks (37.0% vs. 37.2%, *p* = 0.969), <32 weeks (54.6% vs. 52.3%, *p* = 0.684), <34 weeks (64.8% vs. 60.7%, *p* = 0.455), and <37 weeks (77.8% vs. 73.8%, *p* = 0.417) of gestation, as well as the rate of preterm premature rupture of membranes (26.9% vs. 22.5%, *p* = 0.360), were comparable between the two groups. No differences were observed in the neonatal birth weight or 5 min Apgar score between the groups. After balancing baseline characteristics using propensity score matching, all outcome measures remained similar between the two groups. Subgroup analysis of pregnancies with painless cervical dilation ≥ 4 cm also revealed no significant differences in any outcome measures between those who received amnioreduction and those who did not prior to ECC. **Conclusions**: Amnioreduction performed prior to ECC was not associated with adverse pregnancy outcomes based on the endpoints assessed in this cohort. Because procedure-specific complications were not systematically recorded, prospective studies collecting procedure-related safety data are required before amnioreduction can be definitively recommended.

## 1. Introduction

Preterm birth (PTB), defined as delivery before 37 weeks of gestation, poses significant challenges for both parents and clinicians. Notably, the global PTB rate has shown only a minimal downward trend over the past decade [1]. The earlier PTB occurs, the greater the challenges encountered [2]. According to epidemiological studies conducted in China, Australia, and globally, spontaneous PTB (sPTB) before 32 weeks of gestation accounts for the largest proportion of neonatal mortality [3,4,5].

Cervical insufficiency (CI) is defined as the inability of the uterine cervix to sustain a pregnancy in the absence of clinical contractions or labor during the second trimester [6]. Unexpected painless cervical dilation of >2 cm before 28 weeks of gestation is recognized as a typical sign of CI, regardless of whether the pregnancy has a history of sPTB [7]. Emergency cervical cerclage (ECC), also referred to as rescue cerclage, is recommended for both singleton and twin pregnancies with painless cervical dilation in the second trimester [8]. For singleton pregnancies, ECC has been shown to delay delivery by an average of 34 days compared with expectant management or bed rest alone [8]. This prolongation of gestational age (GA) at delivery is valuable, as mortality and morbidity rates in very preterm infants are significantly high [9,10].

A critical first step during ECC is the intact retraction of the protruding amniotic membrane back into the uterine cavity. Managing a tense amniotic membrane that has protruded through a dilated cervix of ≥4 cm into the vagina remains a challenging scenario—even for experienced obstetricians. Amniocentesis is an optional procedure that may be performed prior to ECC, serving two primary purposes: (1) reducing tension in the protruding amniotic sac by extracting a certain amount of amniotic fluid, a process known as amnioreduction [11,12,13], and (2) improving patient selection for rescue cerclage based on amniotic fluid screening [14]. Amnioreduction effectively lowers amniotic sac tension, facilitating the intact retraction of the amniotic membrane back into the uterine cavity, particularly in pregnancies with more severe cervical dilation. However, its effect on pregnancy outcomes has been inconsistent, and previous studies have often involved small sample sizes [11,12,13,15].

To fill this knowledge gap, we conducted a multicenter retrospective cohort study to evaluate the effect of amnioreduction prior to ECC on pregnancy outcomes.

## 2. Material and Methods

### 2.1. Study Design and Participants

This was a retrospective multicenter cohort study conducted across four tertiary hospitals in China: three tertiary general hospitals, including Shanghai East Hospital, Peking University First Hospital, and Sun Yat-sen Memorial Hospital, and one specialized maternity hospital, the Women’s Hospital of Zhejiang University. All high-risk pregnant women with possible indications for ECC were evaluated and reviewed by senior obstetricians using identical assessment criteria. This standardized workflow substantially reduced potential between-center heterogeneity and minimized inter-institutional bias among the 4 study centers. ECC was indicated for women with painless cervical dilation detected between 14 + 0 and 27 + 6 weeks of gestation by physical examination or transvaginal ultrasound, in accordance with the guidelines for cervical cerclage established by the Royal College of Obstetricians and Gynecologists (RCOG) [8].

Contraindications for ECC included: (1) rupture of fetal membranes; (2) clinical chorioamnionitis, diagnosed according to the Gibbs criteria, which require the presence of fever (≥37.8 °C) accompanied by more than two of the following clinical signs: maternal tachycardia (>100 bpm), fetal tachycardia (>160 bpm), leukocytosis (WBC count > 15,000/mm^3^), uterine tenderness, and foul-smelling amniotic fluid [16]; (3) vaginitis, including bacterial vaginosis (Nugent score ≥ 7) [17], or detection of fungus or trichomoniasis via double fluorescent staining; or (4) pregnancies complicated by major fetal anomalies, severe maternal complications, or large subchorionic hematomas. Patients scheduled for surgical treatment who ultimately abandoned the procedure owing to a non-retractable or ruptured amniotic sac were excluded from the present study.

### 2.2. Surgical Treatment and Follow-Up

Transabdominal amnioreduction was considered when the prolapsing amniotic sac was tense and protruded to or beyond the external cervical os, or when cervical dilation was advanced (generally ≥ 4 cm), or when the operating obstetrician judged that intact retraction of the membranes would otherwise be difficult. No fixed numerical threshold was pre-specified, and the final decision rested on the operator’s intraoperative assessment.

For pregnancies that underwent amnioreduction, procedure-attributable events (need for a repeat amnioreduction, bleeding at the puncture site, transient fetal bradycardia, and inadvertent membrane rupture) were rare; however, these complications were not systematically and uniformly documented across the participating centers. The volume removed was individualized rather than standardized by GA. Under continuous transabdominal ultrasound (Mindray UMT-500, Shenzhen, China), the deepest vertical pocket was assessed before and after the procedure, and fluid was withdrawn only to the extent required to decompress the protruding sac while maintaining a normal residual deepest vertical pocket (>2 cm) to avoid oligohydramnios. The volume removed generally ranged from 100 to 400 mL, and the calculated median (interquartile range, IQR) volume in the cohort was 195 (136.3–250.0) mL, which was consistent with reported protocols for therapeutic amnioreduction prior to ECC [12].

ECC was also performed by experienced senior obstetricians at the four institutions immediately following amnioreduction. The surgeries were conducted under local or general anesthesia, as determined by the anesthesiologist. Three 1-0 braided or parallel silk threads (ETHICON SA87G, Shanghai, China), or a Mersilene belt (ETHICON RS22, Shanghai, China), were used as cerclage materials, with 4 to 5 stitches applied. The cerclage materials were positioned as high as possible, and the choice of material and surgical technique (Shirodkar or McDonald) was made by the surgeons. Antibiotics and tocolytics were administered during the surgery and continued for the following 48 h. Complete blood counts and C-reactive protein (CRP) levels were monitored before and after surgery as sensitive indicators for predicting chorioamnionitis. Patients were discharged approximately one week after ECC and received outpatient follow-up every one to two weeks. Vaginal progesterone 200 mg (Utrogestan, Cyndea Pharma SL, Spain) was prescribed routinely one week following ECC for patients with a closed cervix. Nevertheless, several pregnant patients declined this treatment owing to increased vaginal discharge or concerns regarding local drug irritation.

Ultrasonography (Voluson E10, Austria) was conducted by certified sonographers with expertise in fetal medicine, holding qualifications from the Fetal Medicine Foundation. Serial transvaginal ultrasound monitoring was performed to track changes in cervical length (CL) after the surgery. Patients were admitted to the hospital if CL was less than 10 mm or if physical examination revealed cervical dilation. If the GA was less than 34 weeks of gestation, and there was a high risk of imminent delivery within one week, four doses of 6 mg dexamethasone (Southwest Pharmaceutical Co., Ltd., Chongqing, China) were prescribed to induce fetal lung maturation. Magnesium sulfate (Jinyao Pharmaceutical Co., Ltd., Tianjin, China) treatment was administered to reduce the risk of fetal cerebral palsy if labor was initiated before 32 weeks.

### 2.3. Data Collection and Definitions

**Figure 1:**
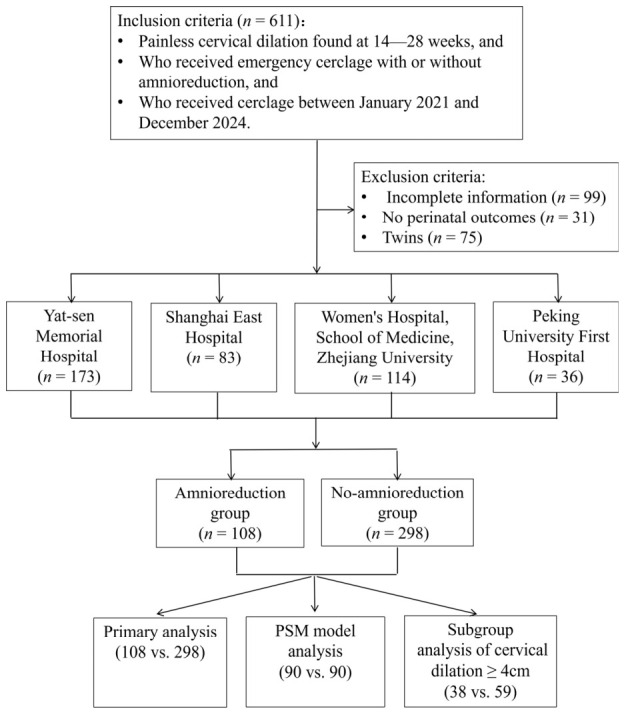
Flowchart of participant enrollment in this retrospective cohort study.

From 1 July 2021 to 31 December 2024, a total of 611 ECC surgeries were registered across the four institutions. After excluding 99 pregnancies with incomplete data or who abandoned the procedure owing to a non-retractable or ruptured amniotic sac (1 case from Shanghai East Hospital, no case from Peking University First Hospital, 3 cases from Sun Yat-sen Memorial Hospital, and 2 cases from Women’s Hospital, Zhejiang University), 31 cases with no perinatal outcomes, and 75 twin pregnancies, a total of 406 singleton pregnancies were included in this study. These were classified into the amnioreduction group (*n* = 108) and the non-amnioreduction group (*n* = 298) according to whether amnioreduction was performed prior to ECC. A flowchart detailing the cohort study is presented in Figure 1.

Detailed maternal risk factors for preterm birth were extracted from electronic medical record systems and included maternal age, gravidity, parity, number of prior pregnancy losses before 14 weeks of gestation, number of prior deliveries between 14 and 34 weeks of gestation, number of hysteroscopic procedures, history of cervical cerclage, polycystic ovary syndrome, size of cervical dilation, number of cerclage stitches, and GA at cerclage. GA at delivery was the primary outcome measure, while other pregnancy outcome indicators included pregnancy latency from cerclage to delivery, rates of preterm birth, PPROM, neonatal birth weight, and 5 min Apgar score were secondary outcome measures. For evaluating the safety of the amnioreduction and cervical cerclage, we compared the rate of delivery within 7 days after surgery, and rate of operation failure between the two groups. Operation failure was defined as delivery within 24 h after surgery, with or without PPROM. GA was determined based on the last menstrual period and confirmed by first-trimester ultrasound measurement of crown-rump length. If there was a discrepancy of more than 7 days, GA was adjusted to match the ultrasound age.

The study received approval from the ethics committees of Shanghai East Hospital (number 2019-195 on 26 June 2019), Sun Yat-sen Memorial Hospital (number SYSEC-KY-KS 2020-024 on 11 March 2020), Peking University First Hospital (number 2016[1259] on 31 May 2017), and the Women’s Hospital of Zhejiang University (number IRB-20200044-R on 21 May 2020). The program for preventing preterm birth among pregnancies complicated by CI was approved by the Pudong New Area Obstetrics Professional Committee. All women with indications for ECC were informed about the risks and potential benefits of the procedure and provided written informed consent prior to surgery. We declare that the investigations were conducted in accordance with the principles outlined in the Declaration of Helsinki (1975, revised in 2013).

### 2.4. Statistical Analysis

Continuous variables with normal distribution were presented as mean ± standard deviation, and compared using independent-samples *t* test. Non-normally distributed continuous variables were expressed as median (IQR), and compared with the Mann–Whitney U test. Categorical variables were shown as number (percentage), and compared using the χ^2^ test or Fisher’s exact test where appropriate. All statistical tests were two-tailed, and a *p*-value < 0.05 was considered statistically significant. Kaplan–Meier survival analysis was used to generate curves for GA at delivery, with intergroup comparisons performed via the log-rank test. The analysis was performed using SPSS software (version 26.0). All statistical tests were two-tailed, and a *p*-value < 0.05 was considered statistically significant.

To address potential confounding risk factors, we constructed a multivariate linear regression model to evaluate factors associated with GA at delivery. Maternal age, gravidity, parity, prior preterm delivery between 14 and 34 weeks of gestation, size of cervical dilation, number of cerclage stitches, and preoperative CRP levels were selected as covariates, as associations between these factors and preterm birth have been reported in previous studies [18,19]. These factors also exhibited differences between the amnioreduction group and the non-amnioreduction group. The assumptions of the linear model were verified via residual statistical analysis and diagnostic tests. Additionally, a propensity score matching (PSM) model was utilized to conduct sensitivity analyses.

As noted in prior research, amnioreduction was considered to be more beneficial for pregnancies with painless cervical dilation ≥ 4 cm, which would likely experience higher tension in the protruded amniotic sac [11]. Therefore, we conducted a subgroup analysis within this population to compare perinatal outcomes between the amnioreduction group and the non-amnioreduction group.

Simple linear regression analysis was performed to explore the linear association between the volume of amnioreduction and GA at delivery. Post hoc analysis was conducted by PASS 2021 software to evaluate the risk of type II error in the PSM model and subgroup analysis.

## 3. Results

### 3.1. Comparison of Maternal Characteristics and Pregnancy Outcomes Between Amnioreduction and Non-Amnioreduction Groups

Compared with pregnancies in the non-amnioreduction group, those in the amnioreduction group exhibited higher maternal age (33.1 ± 4.4 vs. 32.1 ± 4.5, *p* = 0.046), higher gravidity (3 (2, 4) vs. 2 (1, 4), *p* = 0.002), and higher parity (parity 1: 36.1% vs. 25.5%, parity ≥ 2: 5.6% vs. 2.7%, *p* = 0.028). Additionally, there was a greater number of prior deliveries between 14 and 34 weeks of gestation (1–2 deliveries: 38.0% vs. 23.8%, ≥ 3 deliveries: 1.9% vs. 0.7%, *p* = 0.011) and greater cervical dilation (3.0 (2.0, 4.0) vs. 2.0 (1.0, 3.0), *p* < 0.001). The amnioreduction group also had a lower rate of cerclage with 2 stitches (13.0% vs. 22.1%, *p* = 0.040). Preoperative CRP levels in the amnioreduction group were higher than those in the non-amnioreduction group (≥15 mg/L: 18.5% vs. 10.1%, *p* = 0.022). Other indicators, including the number of prior pregnancy losses before 14 weeks of gestation, number of hysteroscopic procedures, history of cervical cerclage, polycystic ovary syndrome, GA at cerclage, and infection indicators (including preoperative leukocyte and neutrophil levels) were similar between the two groups. Detailed results are presented in Table 1.

**Figure 2:**
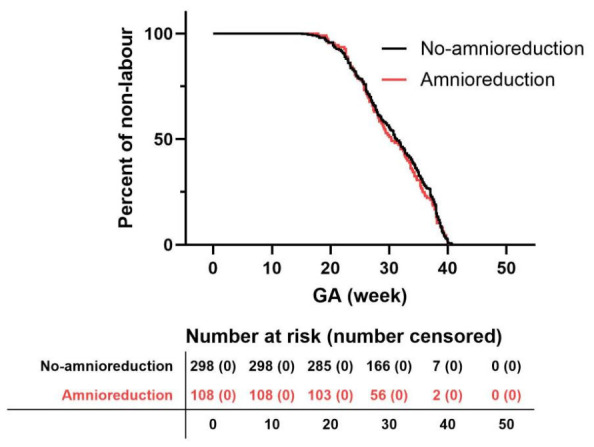
Kaplan–Meier curves of GA at delivery classified by amnioreduction status (*n* = 406).

Table 2 outlines the maternal and neonatal outcome indicators in the two groups. The GA at delivery was similar between the groups (30.4 (25.7, 35.9) vs. 31.2 (26.0, 37.0) weeks, *p* = 0.655), as was pregnancy latency 49.0 (15.0, 87.5) vs. 57.0 (18.0, 92.3) days, *p* = 0.397). The rates of preterm birth at <28, <32, <34, and <37 weeks of gestation, as well as the rate of PPROM, were all comparable between the amnioreduction and non-amnioreduction groups. Neonatal birth weight and 5 min Apgar scores were also similar between the two groups. The Kaplan–Meier curves for GA at delivery did not differ significantly between the two groups (log-rank test, χ^2^ = 0.215, *p* = 0.643) (Figure 2). 

### 3.2. Effect of Amnioreduction on GA at Delivery in Pregnancies with Painless Cervical Dilation

We employed a multivariate linear regression model to evaluate the effect of covariates on GA at delivery in the cohort of pregnancies, with detailed results presented in Table 3. The mean residual value of zero confirmed linearity, and the Durbin–Watson test (1.786) and the range of standardized residuals (−2.504 to 1.574) supported homoscedasticity. The large sample size (*n* = 406) ensured the robustness of residual normality based on the central limit theorem, and variance inflation factor (VIF = 1.000) and condition index (1.769) excluded multicollinearity. No material violations were identified.

The results indicated that amnioreduction had an insignificant influence on GA at delivery (β = 0.037, 95% CI: −0.069, 0.143 weeks). However, parity of ≥2 was associated with an increased GA at delivery (β = 0.251, 95% CI: 0.009, 0.492 weeks), while the size of cervical dilation was associated with a decreased GA at delivery (β = −0.028, 95% CI: −0.049, 0.006 weeks).

Simple linear regression analysis was performed to explore the linear association between the volume of amnioreduction and GA at delivery. The unstandardized regression coefficient was 0.007 (standardized β = 0.100), which indicated that each 1 mL increase in aspirated amniotic fluid was numerically associated with a 0.007-week prolongation in GA at delivery, but this linear correlation was not statistically significant (t = 1.033, *p* = 0.304).

### 3.3. Construction of PSM Model for Sensitivity Analysis

To balance the covariates that differed between the amnioreduction and non-amnioreduction groups, we constructed a PSM model cohort, which included 90 pregnancies in the amnioreduction group and 90 pregnancies in the non-amnioreduction group. Eight covariates—maternal age, gravidity, parity, number of prior deliveries between 14 and 34 weeks of gestation, size of cervical dilation, number of cerclage stitches, GA at cerclage and CRP levels were balanced in the PSM model, as these factors had potential associations with pregnancy outcomes. Detailed information is presented in Table S1.

After balancing the covariates between the amnioreduction and non-amnioreduction groups, we compared maternal and neonatal outcomes between the two groups. The GA at delivery was similar (30.6 (26.1, 36.6) vs. 28.6 (25.2, 34.5) weeks, *p* = 0.101), as was the pregnancy latency from cerclage to delivery (54.5 (15.0, 88.3) vs. 41.5 (10.8, 75.8) days, *p* = 0.082). The rates of preterm birth were also comparable: < 28 weeks (35.6% vs. 45.6%, *p* = 0.172), <32 weeks (54.4% vs. 63.3%, *p* = 0.226), <34 weeks (61.1% vs. 72.2%, *p* = 0.114), and <37 weeks (75.6% vs. 84.4%, *p* = 0.136) of gestation. The incidence of PPROM before 34 weeks of gestation was similar between the two groups (25.6% vs. 30.0%, *p* = 0.506).

To evaluate neonatal outcomes, we compared neonatal birth weight and 5 min Apgar scores between the amnioreduction and non-amnioreduction groups. The median (IQR) neonatal birth weight and 5 min Apgar scores were similar between the two groups (Table 4).

Post hoc power analysis was performed for this cohort of 90 patients in each group to quantify the risk of type II error. Based on the median (IQR) GA: 28.6 (25.2, 34.5) weeks in the no-amnioreduction group and 30.6 (26.1, 36.6) weeks in the amnioreduction group (since GA at delivery as the primary outcome measure). The mean difference was estimated to be 2.0 weeks with a pooled standard deviation of 7.33, yielding a standardized mean difference of 0.273. With a two-sided α of 0.05 and equal sample size of 90 per group, the post hoc statistical power was only 42.3%, which was substantially below the conventional threshold of 80%.

### 3.4. Subgroup Analysis on the Effect of Amnioreduction Prior to ECC in Pregnancies with Painless Cervical Dilation ≥ 4 cm

The influence of ECC on pregnancies with painless cervical dilation ≥ 4 cm is controversial. Therefore, we conducted a subgroup analysis of pregnancies with painless cervical dilation ≥ 4 cm, comparing those who received amnioreduction prior to ECC with those who did not. The results indicated no significant differences in maternal characteristics, which are detailed in Table S2.

Pregnancy outcomes for pregnancies with cervical dilation sizes of ≥ 4 cm (*n* = 97) are described in Table 5. We compared the pregnancy outcomes between those who received amnioreduction (*n* = 38) and those who did not (*n* = 59) prior to ECC. The mean (SD) GA at cerclage was 22.3 (2.3) weeks, while the median (IQR) GA at delivery was 28.1 (24.0, 33.2) weeks. The rates of preterm birth were 54.6% for <28 weeks, 70.1% for <32 weeks, 78.4% for <34 weeks, and 86.6% for <37 weeks of gestation, respectively. The median (IQR) birth weight was 1135.0 (650.0, 2300.0) g, and 56.7% of neonates had a 5 min Apgar score ≥ 7. There were no significant differences in outcome indicators between pregnancies that received amnioreduction and those that did not prior to ECC.

Post hoc power analysis was conducted for this subgroup (38 patients in the amnioreduction group and 59 patients in the no-amnioreduction group) to assess the risk of type II error. Based on the median (IQR) GA: 28.0 (24.0, 33.2) weeks in the amnioreduction group versus 26.7 (23.0, 34.1) weeks in the no-amnioreduction group, the estimated mean difference was 1.3 weeks with a pooled standard deviation of 7.52, corresponding to a standardized mean difference of 0.173. Under the setting of two-sided α = 0.05 for independent samples t-test, the post hoc statistical power was only 15.7%, which was far below the conventional acceptable threshold of 80%.

## 4. Discussion

Our retrospective cohort study demonstrated that pregnancy outcome measures, including GA at delivery, pregnancy latency, rates of preterm birth, and PPROM, were similar between the amnioreduction group and the non-amnioreduction group, both in the general population and in the subgroup of pregnancies with cervical dilation ≥ 4 cm. Based on these findings, we conclude that amnioreduction performed before ECC was not associated with adverse pregnancy outcomes in this cohort. These findings should be regarded as hypothesis-generating and support the need for a randomized controlled trial while providing a clinical reference for obstetricians.

ECC is a technically challenging procedure, particularly when cervical dilation exceeds 4 cm and fetal membranes have prolapsed into or beyond the cervical canal. Various techniques have been described to facilitate the retraction of prolapsed fetal membranes back into the uterine cavity and to maintain their position above the cervical suture site. These techniques include positioning the patient in the Trendelenburg position (with or without instilling 250 mL of normal saline into the bladder) or using an inflated Foley catheter balloon [20,21].

Recent clinical evidence has gradually validated the clinical utility of pre-cerclage amnioreduction for patients presenting with advanced cervical dilation and bulging fetal membranes before emergency cerclage [11,12,13,15]. Consistent with our exploratory findings in singleton pregnancies, a recent retrospective cohort study focusing on twin gestations also indicated that amnioreduction prior to emergency cerclage could acquire significantly prolonged gestational latency and improved neonatal survival, even among patients with a fully dilated cervix and prolapsed amniotic membranes [22]. Notably, twin pregnancies are characterized by higher intrauterine pressure and greater cervical mechanical tension, which brings greater technical challenges to intraoperative fetal membrane retraction. Accordingly, the decompressive advantage of amnioreduction tends to be more pronounced in multiple gestations, which broadens the eligible population of this adjunct surgical strategy beyond singleton cohorts. Beyond evaluating the direct therapeutic efficacy of amnioreduction, recent research has focused on optimizing preoperative patient selection to improve the success rate of emergency cerclage. One prospective observational study reported that concurrent measurement of amniotic fluid IL-6 during amnioreduction, where elevated intra-amniotic IL-6 levels (≥3000 pg/mL) could effectively screen for subclinical intra-amniotic inflammation and identify patients who were unlikely to benefit from rescue cerclage [14]. Collectively, current evidence indicate that pre-cerclage amnioreduction may be a feasible adjunct technique for emergency cerclage across both singleton and twin pregnancies.

In contrast to our findings, Subeen Hong et al. reported that pre-emergency cerclage amnioreduction was correlated with unfavorable maternal and neonatal pregnancy outcomes [15]. In their PSM study consisting of 25 matched pairs, participants in the amnioreduction group delivered at a significantly earlier GA (24.8 ± 5.2 weeks) and had a substantially shorter interval from cerclage to delivery (3.1 ± 3.5 weeks). These outcomes differ markedly from our results, where the median delivery GA was 30.4 (25.7, 35.9) weeks and the median cerclage-to-delivery interval was 49.0 (15.0, 87.5) days, as well as the previously reported median interval of 34 days [8]. Several potential sources of heterogeneity may explain these inconsistent results across studies. GA range at cervical cerclage was higher in our study (22.8 ± 2.5 weeks in amnioreduction group and 23.0 ± 2.2 weeks in no-amnioreduction group) than Subeen Hong et al.’s (21.6 ± 2.5 and 21.5 ± 2.6 weeks, respectively) in the PSM model. Furthermore, small sample size in Subeen Hong et al.’s study was more easy to introduce selection bias and further amplify between-study discrepancies. Of note, the rate of surgical failure rate, which was defined as delivery within 24 h, was 24% among patients who underwent amniocentesis, versus 12.0% in the non-amniocentesis cohort in Subeen Hong et al.’ s study. In our multi-center study, the rate of treatment failure was 0.3% in no-amnioreduction group and 0 in amnioreduction group, which was significantly lower than those in Subeen Hong et al.’ s study. The difference between the two studies indicated that different patient selection may be associated with different pregnancy outcomes.

Given the retrospective nature of this study, we cannot fully clarify the causal effect of pre-cerclage amnioreduction on pregnancy outcomes. However, although the amnioreduction group presented multiple baseline risk factors that were unfavorable to pregnancy prognosis (greater cervical dilation, higher CRP, higher gravidity and parity), it achieved comparable pregnancy outcomes to the no-amnioreduction group. This pattern is compatible either with residual confounding by indication or with a genuine beneficial effect of amnioreduction that offsets the higher baseline risk. The numerically longer pregnancy latency and higher GA at delivery in the amnioreduction group in the PSM cohort and subgroup of pregnancies with cervical dilation ≥ 4 cm were presented as a hypothesis-generating observation that motivates a randomized trial to explore the potential benefit for amnioreduction prior to emergency cerclage. Further, we conducted post hoc power analysis for subgroup analysis in PSM cohort and pregnancies whose cervical dilation ≥ 4 cm. The high probability of type II error indicated that the non-significant between-group difference in this small subgroup was highly likely attributed to insufficient sample size rather than true therapeutic equivalence. Thus, these negative findings should only be interpreted as hypothesis-generating evidence rather than definitive conclusions of no clinical benefit.

In our study, multivariate linear regression analysis indicated that parity ≥ 2 was associated with increased GA at delivery in pregnancies with painless cervical dilation. This finding aligns with the widely held notion that the uterine cavity is larger during a woman’s later pregnancies compared to her first pregnancy [23]. It is plausible that multiparity is associated with a more favorable cervical response to cerclage, possibly due to greater myometrial compliance and a more accommodating uterine environment that buffers against mechanical stress on the incompetent cervix [23]. Additionally, the size of cervical dilation was independently associated with decreased GA at delivery, consistent with Chen C et al.’ s report in 2024, who demonstrated that cervical dilation was linked to a significantly elevated risk of extremely preterm birth (RR 1.98, 95% CI 1.46–2.69, *n* = 141) following emergency cervical cerclage [24]. Terkildsen et al. similarly found that cervical dilation > 3 cm was associated with a reduced likelihood of delivery at or beyond 28 weeks [25]. These convergent findings underscore that cervical dilation at the time of cerclage remains a key determinant of pregnancy latency, independent of whether amnioreduction is performed. After accounting for these covariates, amnioreduction showed no significant association with GA at delivery, further reinforcing the safety profile of the procedure. Taken together, these data suggest that amnioreduction may be offered to patients with advanced cervical dilation (≥4 cm) to facilitate membrane retraction, without expectation of worsening obstetric outcomes, while awaiting confirmation from randomized controlled trials.

Our study has several strengths. Firstly, we included 611 pregnancies from four tertiary medical institutions in the cohort study, with 406 pregnancies analyzed, representing a substantial sample size. Secondly, the results were supported by multivariate linear regression analysis and the construction of the PSM model, which consolidates our conclusions. Thirdly, we conducted a subgroup analysis to provide reference for the effect of amnioreduction prior to emergency cervical cerclage in pregnancies with painless cervical dilation ≥ 4 cm.

Several limitations of our study merit consideration. The primary limitation arises from its retrospective nature. Differences in maternal characteristics between the two groups may have introduced bias. Although we have used multivariable linear regression model and PSM to balance the difference, the limited number of cohort pregnancy was not powered to detect these between-group differences as statistically significant: with 90 pairs, the study had limited power to declare differences of this magnitude significant given the wide dispersion of pregnancy latency. The second limitation is unmeasured confounding factors including surgeon experience, center-level differences in protocol, the unfixed numerical threshold for amnioreduction including tense level of the prolapsing amniotic sac, if the amnion sac protruded to or beyond the external cervical os, and other intraoperative findings were not fully captured. Third, the incidences of procedure-attributable events including the need for a repeat amnioreduction, bleeding at the puncture site, transient fetal bradycardia, and inadvertent membrane rupture were not systematically and uniformly documented across the participating centers. Consequently, procedure-specific complication rates could not be analyzed and are addressed as a limitation. Last but not least, neonatal outcome measures were limited to birth weight and the 5 min Apgar score. Important neonatal complications, including neonatal sepsis, respiratory distress syndrome, intraventricular hemorrhage, and necrotizing enterocolitis, were not analyzed because these data were incomplete. The lack of comprehensive safety endpoints therefore restricted our capacity to conduct a thorough assessment of the procedural safety in this study.

## 5. Conclusions

Amnioreduction prior to emergency cervical cerclage was not associated with adverse pregnancy outcomes in this cohort and may be considered a feasible adjunct to facilitate membrane retraction. However, prospective studies with comprehensive procedure-specific safety data are needed before definitive conclusions can be drawn.

## Figures and Tables

**Table 1:** Baseline maternal clinical characteristics stratified by amnioreduction status (*n* = 406).

Variables	No-Amnioreduction Group (*n* = 298)	Amnioreduction Group (*n* = 108)	*p*-Value
Maternal age (years), mean ± SD	32.1 ± 4.5	33.1 ± 4.4	0.046
Gravidity, median (IQR)	2 (1, 4)	3 (2, 4)	0.002
Parity, *n* (%)			0.028
0	214 (71.8%)	63 (58.3%)	
1	76 (25.5%)	39 (36.1%)	
≥2	8 (2.7%)	6 (5.6%)	
Prior early pregnancy loss (<14 weeks), *n* (%)			0.392
0	145 (48.7%)	48 (44.4%)	
1–2	111 (37.2%)	48 (44.4%)	
≥3	42 (14.1%)	12 (11.1%)	
Prior preterm delivery (14–34 weeks), *n* (%)			0.011
0	225 (75.5%)	65 (60.2%)	
1–2	71 (23.8%)	41 (38.0%)	
≥3	2 (0.7%)	2 (1.9%)	
History of hysteroscopic surgery, *n* (%)			0.177
0	178 (59.7%)	73 (67.6%)	
1–2	87 (29.2%)	29 (26.9%)	
≥3	33 (11.1%)	6 (5.6%)	
Prior cervical cerclage history, *n* (%)	16 (5.4%)	7 (6.5%)	0.668
PCOS, *n* (%)	47 (15.8%)	17 (15.7%)	0.994
Volume of reduced amniotic fluid (mL), median (IQR)	-	195 (136.3–250.0)	
Size of cervical dilation (cm), median (IQR)	2.0 (1.0–3.0)	3.0 (2.0–4.0)	<0.001
Number of cerclage stitches, *n* (%)			0.040
1	232 (77.9%)	94 (87.0%)	
2	66 (22.1%)	14 (13.0%)	
GA at cerclage (weeks), median (IQR)	22.6 (21.1, 24.3)	23.0 (21.7, 24.3)	0.336
Preoperative leukocytes count ≥15 × 10^9^/L, *n* (%)	13 (4.4%)	9 (8.3%)	0.118
Preoperative neutrophils percent ≥ 85%, *n* (%)	37 (12.4%)	10 (9.3%)	0.380
Preoperative CRP level ≥ 15 mg/L, *n* (%)	30 (10.1%)	20 (18.5%)	0.022

CRP, C-reactive protein; GA, gestational age; IQR, interquartile range; PCOS, poly-cystic ovarian syndrome.

**Table 2:** Maternal and neonatal pregnancy outcomes stratified by amnioreduction status.

Outcome Indicators	No-Amnioreduction Group (*n* = 298)	Amnioreduction Group (*n* = 108)	*p*-Value
GA at delivery (weeks), median (IQR)	31.2 (26.0–37.0)	30.4 (25.7–35.9)	0.655
Pregnancy latency (days), median (IQR)	57.0 (18.0–92.3)	49.0 (15.0–87.5)	0.397
Preterm birth, *n* (%)			
<28 weeks of gestation	111 (37.2%)	40 (37.0%)	0.969
<32 weeks of gestation	156 (52.3%)	59 (54.6%)	0.684
<34 weeks of gestation	181 (60.7%)	70 (64.8%)	0.455
<37 weeks of gestation	220 (73.8%)	84 (77.8%)	0.417
PPROM < 34 weeks of gestation, *n* (%)	67 (22.5%)	29 (26.9%)	0.360
Delivery within 7 days after surgery	30 (10.1%)	9 (8.3%)	0.598
Treatment failure, *n* (%)	1 (0.3)	0 (0.0)	>0.999
Neonatal birth weight (g), median (IQR)	1702.5 (830.0–2763.8)	1535.0 (805.0–2670.0)	0.512
5–min Apgar score, *n* (%)			0.446
0	75 (25.2%)	33 (30.6%)	
1–6	6 (2.0%)	1 (0.9%)	
≥7	217 (72.8%)	74 (68.5%)	

GA, gestational age; IQR, interquartile range; PPROM, prelabor premature rupture of membranes.

**Table 3:** Multivariate linear regression model for risk factors associated with GA at delivery (*n* = 406).

Variables	β	95%CI	*p*-Value
Amnioreduction			
No (Reference)	0.000	Reference	
Yes	0.037	−0.069–0.143	0.492
Maternal age (years)	−0.004	−0.013–0.005	0.353
Gravidity	−0.007	−0.039–0.024	0.647
Parity			
0 (Reference)	0.000	Reference	
1	−0.012	−0.124–0.099	0.828
≥2	0.251	0.009–0.492	0.042
Prior preterm delivery (14–34 weeks)			
0 (Reference)	0.000	Reference	
1–2	−0.008	−0.102–0.086	0.873
Size of cervical dilation (cm)	−0.028	−0.049–−0.006	0.011
Number of cerclage stitches			
1 (Reference)	0.000	Reference	
2	−0.071	−0.191–0.049	0.248
Preoperative CRP ≥ 15 mg/L	0.057	−0.088–0.202	0.444

CRP, C-reactive protein. β, unstandardized regression coefficient; 95% CI, 95% confidence interval. This multivariate generalized linear model was adjusted for all covariates listed in the table. Model goodness-of-fit was evaluated via the Pearson chi-square statistic (89.282, df = 76), with the ratio of Pearson chi-square to degrees of freedom equal to 1.175, indicating no overdispersion and acceptable model fit. The Akaike Information Criterion (AIC) of the final model was 568.111, and the Bayesian Information Criterion (BIC) was 592.654. The overall statistical significance of each covariate was assessed using Type III Wald chi-square tests.

**Table 4:** Sensitivity analysis: Maternal and neonatal pregnancy outcomes after 1:1 propensity score matching (PSM) stratified by amnioreduction status.

Outcome Indicators	No-Amnioreduction Group (*n* = 90)	Amnioreduction Group (*n* = 90)	*p*-Value
GA at delivery (weeks), median (IQR)	28.6 (25.2, 34.5)	30.6 (26.1, 36.6)	0.101
Pregnancy latency interval (days), median (IQR)	41.5 (10.8, 75.8)	54.5 (15.0, 88.3)	0.082
Preterm birth, *n* (%)			
<28 weeks of gestation	41 (45.6%)	32 (35.6%)	0.172
<32 weeks of gestation	57 (63.3%)	49 (54.4%)	0.226
<34 weeks of gestation	65 (72.2%)	55 (61.1%)	0.114
<37 weeks of gestation	76 (84.4%)	68 (75.6%)	0.136
PPROM < 34 weeks of gestation, *n* (%)	27 (30.0%)	23 (25.6%)	0.506
Mode of delivery, *n* (%)			0.039
Vaginal delivery	54 (60.0%)	67 (74.4%)	
Cesarean section	36 (40.0%)	23 (25.6%)	
Neonatal birth weight (g), median (IQR)	1390.0 (660.0, 2250.0)	1630.0 (843.8, 2685.0)	0.106
5 min Apgar score, *n* (%)			0.505
0	30 (33.3%)	27 (30.0%)	
1–6	3 (3.3%)	1 (1.1%)	
≥7	57 (63.3%)	62 (68.9%)	

GA, gestational age; IQR, interquartile range; PPROM, preterm premature rupture of membrane.

**Table 5:** Subgroup analysis of pregnancy outcomes among patients with preoperative cervical dilation ≥ 4 cm.

Outcome Indicators	Total Pregnancies (*n* = 97)	No-Amnioreduction Group (*n* = 59)	Amnioreduction Group (*n* = 38)	*p*-Value
GA at delivery (weeks), median (IQR)	28.1 (24.0, 33.2)	26.7 (23.0, 34.1)	28.0 (24.0, 33.2)	0.564
Pregnancy latency (days), median (IQR)	33.0 (10.8, 73.3)	19.0 (8.0, 71)	33.0 (10.8, 73.3)	0.745
Preterm birth, *n* (%)				
<28 weeks of gestation	53 (54.6%)	35 (59.3%)	18 (47.4%)	0.248
<32 weeks of gestation	68 (70.1%)	41 (69.5%)	27 (71.1%)	0.870
<34 weeks of gestation	76 (78.4%)	44 (74.6%)	32 (84.2%)	0.261
<37 weeks of gestation	84 (86.6%)	49 (83.1%)	35 (92.1%)	0.201
PPROM < 34 weeks of gestation, *n* (%)	27 (27.8%)	16 (27.1%)	11 (28.9%)	0.844
Mode of delivery, *n* (%)				0.536
Vaginal delivery	76 (78.4%)	45 (76.3%)	31 (81.6%)	
Cesarean section	21 (21.6%)	14 (23.7%)	7 (18.4%)	
Neonatal birthweight (g), median (IQR)	1135.0 (650.0, 2300.0)	990.0 (510.0, 2120.0)	1135.0 (637.5, 2302.5)	0.564
5 min Apgar score, *n* (%)				0.431
0	41 (42.3%)	26 (44.1%)	15 (39.5%)	
1–6	1 (1.0%)	0 (0.0%)	1 (2.6%)	
≥7	55 (56.7%)	33 (55.9%)	22 (57.9%)	

GA, gestational age; IQR, interquartile range; PPROM, preterm premature rupture of membrane.

## Data Availability

Data are available from the corresponding authors upon reasonable request and with permission of the 4 hospitals.

## Supplementary Materials

The following supporting information can be downloaded at https://www.mdpi.com/article/10.3390/jcm15145431/s1: Table S1. Baseline maternal clinical characteristics after 1:1 PSM classified by amnioreduction status *n* = 180); Table S2. Subgroup analysis: Baseline maternal clinical characteristics of pregnant women with cervical dilation ≥ 4 cm in the pregnancy cohort *n* = 97); Table S3. Cox proportional-hazards model in evaluating risk factors for GA at delivery in the cohort pregnancies (*n* = 406).

## References

[1] Ohuma E.O., Moller A.B., Bradley E., Chakwera S., Hussain-Alkhateeb L., Lewin A., Okwaraji Y.B., Mahanani W.R., Johansson E.W., Lavin T. (2023). National, regional, and global estimates of preterm birth in 2020, with trends from 2010: A systematic analysis. Lancet.

[2] Lehtonen L., Gimeno A., Parra-Llorca A., Vento M. (2017). Early neonatal death: A challenge worldwide. Semin. Fetal Neonatal Med..

[3] Zhang X., Chen X., Li B., Xia L., Zhang S., Ding W., Gao L., Liu A., Xu F., Zhang R. (2022). Changes in the live birth profile in Henan, China: A hospital registry-based study. Birth.

[4] Barros F.C., Vélez Mdel P. (2006). Temporal trends of preterm birth subtypes and neonatal outcomes. Obs. Gynecol..

[5] Tindal K., Bimal G., Flenady V., Gordon A., Farrell T., Davies-Tuck M. (2022). Causes of perinatal deaths in Australia: Slow progress in the preterm period. Aust. N. Z. J. Obs. Gynaecol..

[6] The American College of Obstetricians and Gynecologists (2014). ACOG Practice Bulletin No.142: Cerclage for the management of cervical insufficiency. Obs. Gynecol..

[7] Ehsanipoor R.M., Seligman N.S., Saccone G., Szymanski L.M., Wissinger C., Werner E.F., Berghella V. (2015). Physical Examination-Indicated Cerclage: A Systematic Review and Meta-analysis. Obs. Gynecol..

[8] Shennan A.H., Story L. (2022). Royal College of Obstetricians Gynaecologists Cervical Cerclage: Green-top Guideline No 75. BJOG.

[9] Patel R.M. (2016). Short- and Long-Term Outcomes for Extremely Preterm Infants. Am. J. Perinatol..

[10] Cao Y., Jiang S., Sun J., Hei M., Wang L., Zhang H., Ma X., Wu H., Li X., Sun H. (2021). Assessment of Neonatal Intensive Care Unit Practices, Morbidity, and Mortality Among Very Preterm Infants in China. JAMA Netw. Open.

[11] Proctor L.K., Ronzoni S., Melamed N., Nevo O., Cohen H., Barrett J. (2022). Amnioreduction with rescue cerclage at advanced cervical dilation or gestational age. J. Matern. Fetal Neonatal Med..

[12] Zhang Y., Wang Q., Tan Z., Zhou J., Zhang P., Hou H., Yin Y., Han Z. (2022). The Role of Amnioreduction in Emergency Cervical Cerclage with Bulging Membranes: A Retrospective Comparative Study. Front. Surg..

[13] Locatelli A., Vergani P., Bellini P., Strobelt N., Arreghini A., Ghidini A. (1999). Amnioreduction in emergency cerclage with prolapsed membranes: Comparison of two methods for reducing the membranes. Am. J. Perinatol..

[14] Werlang A., De Simone A., Jones G. (2025). Amniocentesis and Therapeutic Amnioreduction Before Rescue Cerclage: Improving Patient Selection for Rescue Cerclage based on Amniotic Fluid Screening. J. Obs. Gynaecol. Can..

[15] Hong S., Ko H.S., Kim S., Jo Y.S., Park I.Y. (2023). Effects of Amnioreduction before Physical Examination-Indicated Cerclage on Pregnancy Outcomes: A Propensity Score Matched Study. J. Clin. Med..

[16] Gibbs R.S., Blanco J.D., St Clair P.J., Castaneda Y.S. (1982). Quantitative bacteriology of amniotic fluid from women with clinical intraamniotic infection at term. J. Infect. Dis..

[17] Nugent R.P., Krohn M.A., Hillier S.L. (1991). Reliability of diagnosing bacterial vaginosis is improved by a standardized method of Gram stain interpretation. J. Clin. Microbiol..

[18] Chen C., Guo S., Fan C., Gao F. (2024). Nomogram-based risk assessment for emergency cervical cerclage failure in patients with cervical insufficiency. Heliyon.

[19] Xu Z.M., Zhang J., Hong X.L., Liu J., Yang Z.Z., Pan M. (2023). Comparison of two stitches versus one stitch for emergency cervical cerclage to prevent preterm birth in singleton pregnancies. Int. J. Gynaecol. Obstet..

[20] Scheerer L.J., Lam F., Bartolucci L., Katz M. (1989). A new technique for reduction of prolapsed fetal membranes for emergency cervical cerclage. Obs. Gynecol..

[21] Son G.-H., Chang K.H.-J., Song J.-E., Lee K.-Y. (2015). Use of a uniconcave balloon in emergency cerclage. Am. J. Obs. Gynecol..

[22] Yalınkaya A., Oğlak S.C., Gündüz R., Yılmaz E.Z., Bolluk G., Yayla M. (2025). Outcomes of emergency cervical cerclage after amnioreduction in twin pregnancies with a fully dilated cervix and amniotic membrane prolapse. J. Turk. Ger. Gynecol. Assoc..

[23] Sørnes T., Bakke T. (1989). Uterine size, parity and umbilical cord length. Acta Obs. Gynecol. Scand..

[24] Chen C., Zhao B., Pan Y., Chen L., Yang X., Lv M., Qiu L., Yang M., Ying X., Wang M. (2024). Development and validation of models for predicting preterm birth and pregnancy latency following emergency cervical cerclage: A multicenter cohort study. Acta Obs. Gynecol. Scand..

[25] Terkildsen M.F., Parilla B.V., Kumar P., Grobman W.A. (2003). Factors associated with success of emergent second-trimester cerclage. Obs. Gynecol..

