# Supplementary material for: Effect of Amnioreduction Prior to Emergency Cervical Cerclage on Pregnancy Outcomes in Singleton Pregnancies with Painless Cervical Dilation: A Multicenter Retrospective Study
Source: J Clin Med. 2026 Jul 10;15(14):5431. doi: 10.3390/jcm15145431 (PMC13412723; doi:10.3390/jcm15145431)
Supplement: Supplementary file 1 [file jcm-15-05431-s001.zip › jcm-4356780-supplementary.pdf]

Table S1. Baseline maternal clinical characteristics after 1:1 propensity score matching (PSM) stratified by amnioreduction status (n=180)

**Variables**	**No-amnioreduction group (n = 90)**	**Amnioreduction group (n = 90)**	***p* -value**	***Test SMD***
Maternal age (years), mean ± SD	33.0 (4.5)	32.7 (4.4)	0.616	0.075
Gravidity, mean ± SD	2.9 (1.4)	2.9 (1.3)	0.827	0.033
Parity, *n* (%)				
0	59 (65.6%)	55 (61.1%)	0.553	0.163
1	30 (33.3%)	31 (34.4%)		
≥ 2	1 (1.1%)	4 (4.4%)		
Prior early pregnancy loss (<14 weeks), *n* (%)			0.547	
0	41 (45.6%)	41 (45.6%)		
1—2	39 (43.3%)	43 (47.8%)		
≥ 3	10 (11.1%)	6 (6.7%)		
Prior preterm delivery (14–34 weeks), *n* (%)			0.585	0.155
0	59 (65.6%)	58 (64.4%)		
1—2	30 (33.3%)	32 (35.6%)		
≥ 3	1 (1.1%)	0 (0.0%)		
History of hysteroscopic surgery, *n* (%)			0.158	
0	55 (61.1%)	60 (66.7%)		
1—2	24 (26.7%)	26 (28.9%)		
≥ 3	11 (12.2%)	4 (4.4%)		
Prior cervical cerclage history, *n* (%)	6 (6.7%)	3 (3.3%)	0.305	
PCOS, *n* (%)	79 (87.8%)	74 (82.2%)	0.297	
Volume of reduced amniotic fluid (mL), median (IQR)	-	195.0 (140.0, 250.0)		
Size of cervical dilation (cm), mean ± SD	3.3 (2.0)	3.2 (1.9)	0.799	0.038
Number of cerclage stitches, *n* (%)			0.832	0.063
1	76 (84.4%)	78 (86.7%)		
2	14 (15.6%)	12 (13.3%)		
GA at cerclage (weeks), *n* (%)	22.8 (2.5)	23.0 (2.2)	0.771	0.043
Preoperative leukocytes ≥ 15 $\times 10^9$/L, *n* (%)	3 (3.3%)	9 (10.0%)	0.135	

Preoperative neutrophils percent ≥ 85%, *n* (%)	10 (11.1%)	8 (8.9%)	0.619	
Preoperative CRP ≥ 15mg/L, *n* (%)	14 (15.6%)	14 (15.6%)	1.000	<0.001

CRP, C-reactive protein; GA, gestational age; IQR, interquartile range; PCOS, poly-cystic ovarian syndrome; SD, standard deviation

Table S2. Subgroup analysis: Baseline maternal clinical characteristics of pregnant women with cervical dilation ≥ 4 cm in the pregnancy cohort (n =97)

**Variables**	**Total pregnancies (n = 97)**	**No-amnioreduction group (n = 59)**	**Amnioreduction group (n = 38)**	***p*-value**
Maternal age (years), mean ± SD	32.0 (4.1)	31.5 (4.2)	32.7 (4.0)	0.170
Gravidity, median (IQR)	3 (2, 4)	2 (1, 3)	3 (2, 4)	0.080
Parity, *n* (%)				0.183
0	75 (77.3%)	49 (83.1%)	26 (68.4%)	
1	17 (17.5%)	7 (11.9%)	10 (26.3%)	
≥ 2	5 (5.2%)	3 (5.1%)	2 (5.3%)	
Prior early pregnancy loss (< 14 weeks), *n* (%)				0.580
0	45 (46.4%)	25 (42.4%)	20 (52.6%)	
1—2	42 (43.3%)	27 (45.8%)	15 (39.5%)	
≥ 3	10 (10.3%)	7 (11.9%)	3 (7.9%)	
Prior preterm delivery (14–34 weeks), *n* (%)				0.134
0	71 (73.2%)	46 (78.0%)	25 (65.8%)	
1—2	24 (24.7%)	13 (22.0%)	11 (28.9%)	
≥ 3	2 (2.1%)	0 (0.0%)	2 (5.3%)	
History of hysteroscopic surgery, *n* (%)				0.685
0	66 (68.0%)	39 (66.1%)	27 (71.1%)	
1—2	23 (23.7%)	14 (23.7%)	9 (23.7%)	
≥ 3	8 (8.2%)	6 (10.2%)	2 (5.3%)	
Prior cervical cerclage history, *n* (%)	2 (2.1%)	0 (0.0%)	2 (5.3%)	0.151
PCOS, *n* (%)	18 (18.6)	12 (20.3)	6 (15.8%)	0.574
Volume of reduced amniotic fluid (mL), median (IQR)	-	-	222.5 (177.5, 280.0)	
Size of cervical dilation (cm), median (IQR)	5.0 (4.0, 7.0)	5.0 (4.0, 6.0)	5.0 (4.0, 7.0)	0.820
Number of cerclage stitches, *n* (%)				0.238

1	66 (68.0%)	37 (62.7%)	29 (76.3%)	
2	31 (32.0%)	22 (37.3%)	9 (23.7%)	
GA at cerclage (weeks), mean ± SD	22.3 (2.3)	22.2 (2.5)	22.6 (1.9)	0.369
Preoperative leukocytes ≥ 15×10^9/L, *n* (%)	7 (7.2%)	6 (10.2%)	1 (2.6%)	0.161
Preoperative neutrophils percent ≥ 85%, *n* (%)	15 (15.5%)	9 (15.3%)	6 (15.8%)	0.943
Preoperative CRP ≥ 15mg/L, *n* (%)	19 (19.6%)	10 (16.9%)	9 (23.7%)	0.415

CRP, C-reactive protein; GA, gestational age; IQR, interquartile range; PCOS, poly-cystic ovarian syndrome; SD, standard deviation

Table S3 Cox proportional-hazards model in evaluating risk factors for GA at delivery in the cohort pregnancies (n = 406)

Variable	HR (Exp (B))	95% CI for HR	*p*-value
Amnioreduction (reference: no-amnioreduction	0.903	0.714–1.142	0.395
Maternal age	1.007	0.983–1.031	0.584
Gravidity	1.058	0.971–1.153	0.195
Parity = 0	reference	reference	
Parity = 1	1.118	0.880–1.420	0.362
Parity ≥ 2	0.662	0.375–1.166	0.153
No prior deliveries between 14 and 34 weeks of gestation	reference	reference	
Prior 1–2 deliveries between 14 and 34 weeks of gestation	1.306	1.026–1.664	0.03
Prior ≥3 deliveries between 14 and 34 weeks of gestation	4.341	1.397–13.492	0.011
Cervical dilation on admission (per 1 cm increase)	1.212	1.146–1.281	＜0.001
2 cerclage stitches (reference: 1 stitch)	1.288	0.998–1.662	0.052
